# Acute Peptic Ulcer Disease Caused by Non-Helicobacter pylori-Helicobacter

**DOI:** 10.7759/cureus.14993

**Published:** 2021-05-12

**Authors:** Teresa Da Cunha, Murali Dharan

**Affiliations:** 1 Internal Medicine, University of Connecticut Health, Farmington, USA; 2 Gastroenterology, University of Connecticut Health, Farmington, USA

**Keywords:** non-helicobacter pylori-helicobacter, nhph, h. heilmannii, helicobacter, gastric ulcer, peptic ulcer disease

## Abstract

Human infection by Non-*Helicobacter pylori*-Helicobacter is rare and most commonly transmitted through direct contact with animals. The clinical presentation in most cases is chronic epigastric abdominal pain and it usually leads to chronic gastritis. We present an uncommon case of a patient with acute onset abdominal pain secondary to acute peptic ulcer disease caused by *Helicobacter heilmannii* who underwent successful treatment. We also conducted a review of the literature to understand the epidemiology, etiology, clinical presentation, and the best diagnostic and treatment modalities for Non-*H. pylori-*Helicobacter infections.

## Introduction

Peptic ulcer disease caused by *Helicobacter pylori* is a well-described entity and infection with this bacterium is highly prevalent in humans. In contrast, infection by other Helicobacter species is extremely rare with a prevalence of less than 1% [1]. Non-*H. pylori-H*elicobacter (NHPH) were first described in the stomach of animals by Rappin [2]. These Gram-negative rods commonly colonize the stomach of cats and dogs and transmission from pets to humans through direct contact is suggested [3]. Infection with these microorganisms has been associated with chronic gastritis, gastric ulcers, MALT lymphoma, and even gastric cancer [4,5]. Diagnosis can be challenging given the lack of sensitivity and specificity of noninvasive tests that are typically used to detect *Helicobacter pylori*. Moreover, there are no specific treatment guidelines. This case illustrates a very unusual presentation of infection by NHPH in whom prompt diagnosis and treatment led to complete resolution of the disease.

## Case presentation

A 60-year-old female with a medical history of gastroesophageal reflux disease (GERD), hypertension, hyperlipidemia, and psoriasis presented to the emergency room with a complaint of acute onset right upper quadrant pain associated with nausea and vomiting. Nocturnal exacerbation of pain and sleep disturbance was reported. No fever, weight loss, or change of bowel habit was noted. The patient endorsed frequent use of ibuprofen, a non-steroidal anti-inflammatory drug (NSAID), for several weeks prior to presentation for symptoms related to restless leg syndrome and insomnia. She was also taking aspirin 81 mg daily. She was an ex-smoker, drank wine two to three times a week, and consumed one cup of coffee a day.

On physical examination, she was awake and alert, afebrile, blood pressure 102/68 mm Hg, pulse 53 bpm. No scleral icterus or conjunctival pallor was noted. The abdomen was soft with epigastric tenderness on palpation without any appreciable organomegaly or masses. The remainder of her physical examination was unremarkable.

Laboratory results showed a white blood cell count of 7.1 k/µL, hemoglobin/hematocrit 14.1 g/dL and 41.6%, platelet count of 222,000/µL, normal metabolic profile, liver chemistry, and amylase/lipase. An abdominal ultrasound was suggestive of fatty liver with normal-appearing gallbladder and common bile duct. A computed tomography scan of the abdomen revealed edema and thickening of the gastric antrum and possibly the duodenal bulb without intra-abdominal adenopathy. At this time, the abdominal pain was felt to be secondary to peptic ulcer disease.

**Figure 1:**
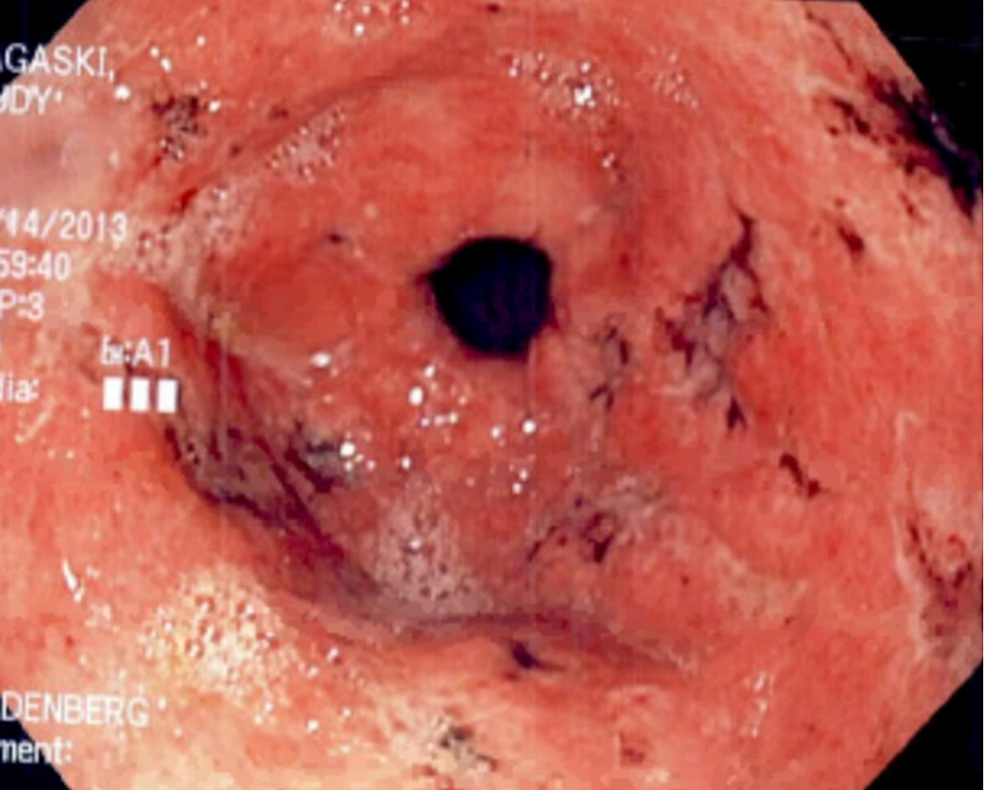
Esophagogastroduodenoscopy showing antral ulcers (some with a dark eschar on the ulcer base) with surrounding inflammation and edema.

**Figure 2:**
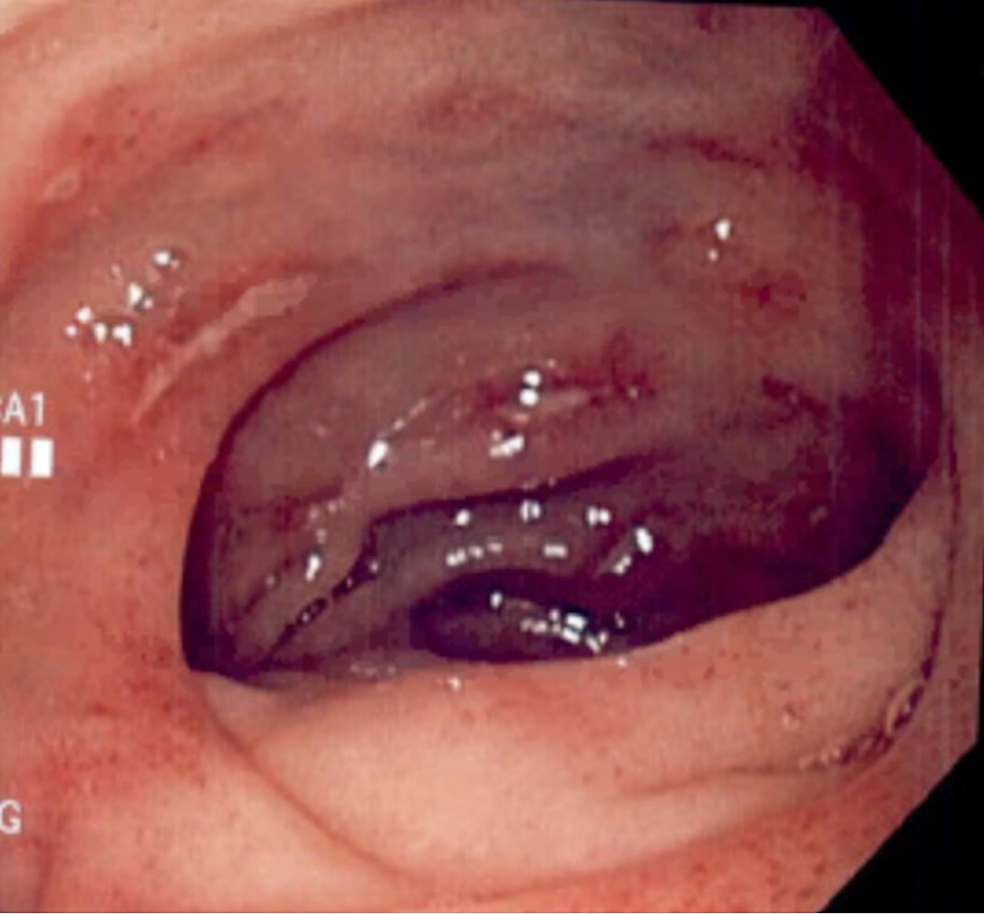
Esophagogastroduodenoscopy showing several linear ulcers in the second portion of the duodenum.

Symptomatic therapy with intravenous hydromorphone, ondansetron, and pantoprazole was initiated. She was subsequently admitted for further work-up. An esophagogastroduodenoscopy (EGD) done on the second day showed multiple superficial non-bleeding antral ulcers with surrounding inflammation and edema. The ulcers were of varying sizes and shapes and some had a dark eschar on the ulcer base (Figure 1). Several linear ulcers were also noted in the second portion of the duodenum (Figure 2). 

**Figure 3:**
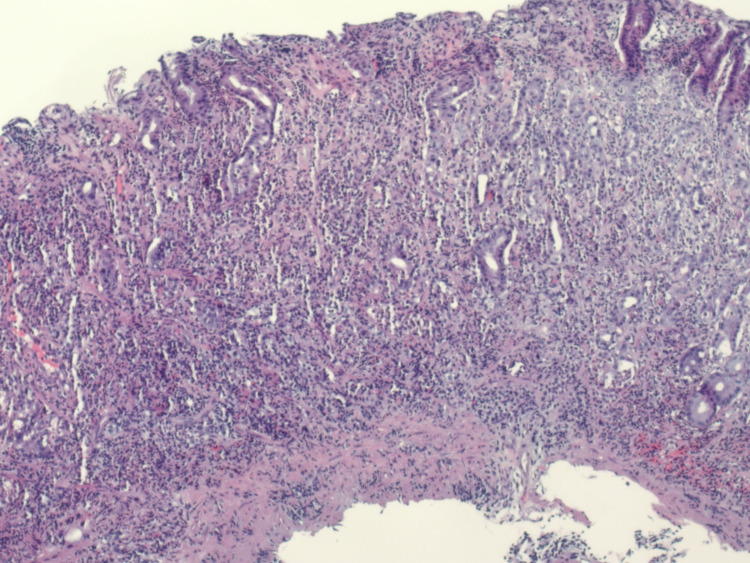
Histopathological findings of the gastric biopsy showing surface erosion of the gastric mucosa with marked acute on chronic inflammation in the lamina propria and marked reactive changes in the gastric epithelium.

**Figure 4:**
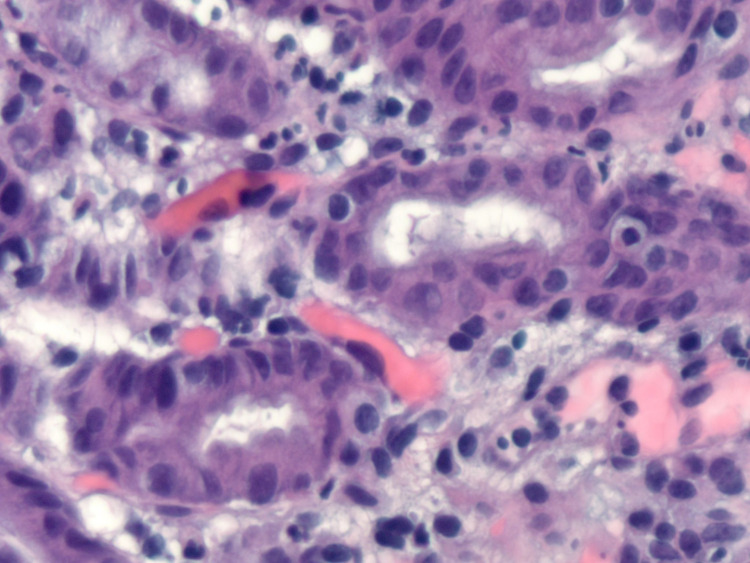
Histopathological findings of the gastric biopsy showing bacteria within the gastric faveolae.

**Figure 5:**
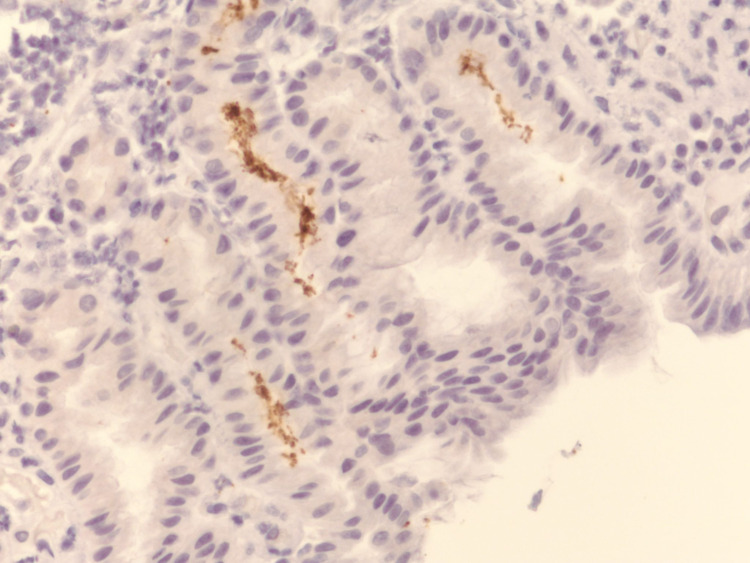
Positive immunohistochemical staining for Helicobacter organisms.

On the third day of admission, her symptoms had significantly improved, and she was discharged home on pantoprazole 40 mg twice daily and famotidine 20 mg tablet at bedtime. Abstinence from NSAID usage was recommended. The histopathological findings of the gastric biopsy showed surface erosion of the gastric mucosa with marked acute chronic inflammation in the lamina propria and marked reactive changes in the gastric epithelium without intestinal metaplasia (Figure 3). Rare organisms were present and immunohistochemical staining (IHC) for Helicobacter organisms was positive, in a pattern consistent with *H. heilmannii* (Figures 4 and 5).

The patient was treated with pantoprazole 40 mg twice daily, amoxicillin 1 g twice daily, and clarithromycin 500 mg twice daily for a total of two weeks. A repeat EGD performed two months later showed complete healing of the stomach and duodenal ulcers and resolution of symptoms.

## Discussion

Helicobacter species are Gram-negative bacteria with a characteristic helical shape. *H. pylori* is the most common pathogen seen in the human gastric mucosa. To date, at least 22 different species have been validated [6]. However, other (non-*H. pylori*) Helicobacter species can also cause gastric disease.

Named *H. heilmannii*, after the German pathologist Konrad Heilmann [7], NHPH organisms were first described in 1987. These microorganisms comprise a group of several species and at least 11 different species have been found in the gastric mucosa of healthy domestic and wild animals [8]. According to its genetic sequence, two main types of *H. heilmannii* can be distinguished. Type 1 is the main Helicobacter species that colonizes the stomach of pigs and is termed *H. suis*. Type 2 is a group of Helicobacter species found predominantly in the stomach of cats and dogs and includes, *H. felis, H. bizzozeronii, *and* H. salomonis.* Lastly, another species that has been detected in the stomachs of several animals and humans but remains uncultivable is termed “Candidatus Helicobacter heilmannii” [9,10]. These organisms are often referred to as HHLO (*H. heilmanii* like organisms).

Unlike *H. pylori*, infection with NHPH species is extremely rare with a reported prevalence of around 0.1-0.5% in Western countries [11]. However, a higher prevalence is reported in the far East (up to 2% in China and 6% in Thailand) [12]. Transmission of these pathogens to humans is thought to occur through direct contact with infected animals [3]. Interestingly, there is at least one case report suggesting transmission of *H. suis* through consumption of infected pork [13]. In the study of Stolte et al., 89% of the patients had contact with animals [3]. In our case, the patient had a very close relationship with her cat.

The clinical symptoms of NHPH are variable. In a study by Heilmann and Borchard [7] comprising of 39 patients, the majority (34) had dyspeptic symptoms, epigastric pain, vomiting, heartburn, and dysphagia. The duration of symptoms ranged from one month to two years. One patient had diarrhea and four were asymptomatic. Acute epigastric abdominal pain as the initial presentation is extremely rare and in addition to our patient, we identified two other cases with a similar presentation. Curiously (similar to our patient), the other cases reported were females in the same age group (sixth decade) and had household cats [14,15]. In our patient, there were other contributing factors to erosive disease of the stomach and duodenum - aspirin and NSAID usage.

The endoscopic findings associated with infection by these bacteria range from normal gastric mucosa, to erythema consistent with chronic gastritis, gastric ulcers, and MALT lymphoma [1,16]. Our patient also had duodenal ulcers which is an uncommon manifestation (although it is unclear if this was due to the infection or NSAID usage). On histopathologic examination, the antrum is the most (and typically the only) affected area and the mononuclear inflammatory infiltrate has a lymphocytic predominance with low neutrophilic activity [17]. The lymphocytic exudation into gastric foveloae appears to be a distinctive feature of NHPH infection and is not seen in *H. pylori* infection. In contrast, epithelial mucus depletion tends to be a feature of *H. pylori* infection and is not seen in NHPH infection [9].

Unlike *H. pylori*, the diagnosis of NHPH is challenging as there are no specific non-invasive tests to accurately identify these bacteria. Antibodies to *H. pylori* may cross-react with “Candidatus H. heilmannii” and *H. suis* thus preventing differentiation. In addition, urease breath test (UBT) has a low sensitivity and can be falsely negative [14]. The NHPH appears to have a lower amount of urease than *H. pylori* and UBT only tends to be positive when the infectious burden is higher [18]. NHPH infection should be considered when UBT is positive but fecal *H. pylori* antigen and serum anti-*H. pylori* immunoglobulin G antibody titers are negative.

The diagnosis of *H. heilmannii* infection is made predominantly by histology using either Giemsa, Steiner, and Whartin-Starry silver stain [11]. Characteristic morphology can be identified - tightly coiled, straight appearing organisms that can measure up to 10 µm in length with bipolar tufts of flagella [8]. A typical “corrugated cigar” or corkscrew appearance has been reported. In comparison, *H. pylori* species appear smaller and more curved. Methylene blue staining yields a more homogenous coloring of NHPH bacteria [9]. The NHPH organisms may be better identified by smears (touch cytology) as they tend to inhabit the mucus layer overlying the surface and foveolar epithelium without the cellular adherence and permeation of the intercellular space noted with *H. pylori* infection [19].

While IHC staining cannot distinguish between NHPH and *H. pylori* species due to cross-reactivity, it helps confirm NHPH infection - based on the morphologic appearance and distribution of the bacteria. In vitro culture of these bacteria is very challenging, given their fastidious nature [11]. The gold standard method for accurate identification and differentiation of these groups of bacteria is polymerase chain reaction assay with the sequencing of specific urease genes [20].

Treatment is recommended for symptomatic patients. Unfortunately, there are no clinical trials directed at NHPH, hence no specific guidelines. However, antibiotic therapy in conjunction with proton-pump inhibitors (typically used for eradication of *H. pylori* infection) has shown satisfactory results in the eradication of NHPH and in many cases, full resolution and healing of the mucosal ulcers and/or gastritis [20]. In our case, the follow-up EGD after treatment showed complete mucosal healing suggesting that the eradication therapy was successful.

## Conclusions

The non-*Helicobacter pylori-H*elicobacter bacterial infection should be considered in patients with dyspepsia and/or erosive disease of the gastric mucosa, especially in patients who have pets (cats and dogs). A high index of suspicion is required and IHC staining or PCR sequencing may be necessary for accurate diagnosis.

## References

[1] Joo M, Kwak JE, Chang SH (2007). Helicobacter heilmannii-associated gastritis: clinicopathologic findings and comparison with Helicobacter pylori-associated gastritis. J Korean Med Sci.

[2] Rappin G (2021). Contribution a l’etude des bacteries de la bouche a l’etat normal. https://www.ncbi.nlm.nih.gov/nlmcatalog/100910029.

[3] Stolte M, Wellens E, Bethke B, Ritter M, Eidt H (1994). Helicobacter heilmannii (formerly Gastrospirillum hominis) gastritis: an infection transmitted by animals?. Scand J Gastroenterol.

[4] Okiyama Y, Matsuzawa K, Hidaka E, Sano K, Akamatsu T, Ota H (2005). Helicobacter heilmannii infection: clinical, endoscopic and histopathological features in Japanese patients. Pathol Int.

[5] Morgner A, Bayerdörffer E, Meining A, Stolte M, Kroher G (1995). Helicobacter heilmannii and gastric cancer. Lancet.

[6] Solnick JV (2003). Clinical significance of Helicobacter species other than Helicobacter pylori. Clin Infect Dis.

[7] Heilmann KL, Borchard F (1991). Gastritis due to spiral shaped bacteria other than Helicobacter pylori: clinical, histological, and ultrastructural findings. Gut.

[8] Baele M, Decostere A, Vandamme P (2008). Isolation and characterization of Helicobacter suis sp. nov. from pig stomachs. Int J Syst Evol Microbiol.

[9] Ierardi E, Monno RA, Gentile A (2001). Helicobacter heilmannii gastritis: a histological and immunohistochemical trait. J Clin Pathol.

[10] Hilzenrat N, Lamoureux E, Weintrub I, Alpert E, Lichter M, Alpert L (2020). Helicobacter heilmannii-like spiral bacteria in gastric mucosal biopsies. Prevalence and clinical significance. Arch Pathol Lab Med.

[11] Bento-Miranda M, Figueiredo C (2014). Helicobacter heilmannii sensu lato: an overview of the infection in humans. World J Gastroenterol.

[12] Ghil HM, Yoo JH, Jung WS, Chung TH, Youn HY, Hwang CY (2009). Survey of Helicobacter infection in domestic and feral cats in Korea. J Vet Sci.

[13] Stolte M, Kroher G, Meining A, Morgner A, Bayerdörffer E, Bethke B (1997). A comparison of Helicobacter pylori and H. heilmannii gastritis. A matched control study involving 404 patients. Scand J Gastroenterol.

[14] Yoshimura M, Isomoto H, Shikuwa S (2002). A case of acute gastric mucosal lesions associated with Helicobacter heilmannii infection. Helicobacter.

[15] Matsumoto T, Kawakubo M, Akamatsu T (2014). Helicobacter heilmannii sensu stricto-related gastric ulcers: a case report. World J Gastroenterol.

[16] Okiyama Y, Matsuzawa K, Hidaka E, Sano K, Akamatsu T, Ota H (2005). Helicobacter heilmannii infection: clinical, endoscopic and histopathological features in Japanese patients. Pathol Int.

[17] Baele M, Pasmans F, Flahou B, Chiers K, Ducatelle R, Haesebrouck F (2009). Non-Helicobacter pylori helicobacters detected in the stomach of humans comprise several naturally occurring Helicobacter species in animals. FEMS Immunol Med Microbiol.

[18] Goji S, Tamura Y, Sasaki M (2015). Helicobacter suis-infected nodular gastritis and a review of diagnostic sensitivity for helicobacter heilmannii-like organisms. Case Rep Gastroenterol.

[19] Van den Bulck K, Decostere A, Baele M (2005). Identification of non-Helicobacter pylori spiral organisms in gastric samples from humans, dogs, and cats. J Clin Microbiol.

[20] Schultz-Süchting F, Stallmach T, Braegger CP (1999). Treatment of Helicobacter heilmannii-associated gastritis in a 14-year-old boy. J Pediatr Gastroenterol Nutr.

